# Self-efficacy in breastfeeding predicts how mothers perceive their preterm infant’s state-regulation

**DOI:** 10.1186/s13006-022-00486-5

**Published:** 2022-06-11

**Authors:** Andreas Karlsson Rosenblad, Eva-Lotta Funkquist

**Affiliations:** 1 Department of Medical Sciences, Division of Clinical Diabetology and Metabolism, Uppsala University, Uppsala, Sweden; 2 Department of Neurobiology, Care Sciences and Society, Division of Family Medicine and Primary Care, Karolinska Institutet, Solna, Sweden; 3 Department of Women’s and Children’s Health, Uppsala University, Dag Hammarskjölds väg 14 B, 752 37 Uppsala, Sweden

**Keywords:** Breastfeeding, Infant, Mothers, Neonatal, Premature, Self-efficacy, State-regulation, Support

## Abstract

**Background:**

Mothers of preterm infants often perceive the infant as having problems with crying, sleeping and feeding, sometimes summarised as ‘state-regulation’. Breastfeeding rates are lower among preterm infants, and the mother’s self-efficacy in breastfeeding is central to understanding which mothers are going to breastfeed their infants. We have previously shown that mothers with higher self-efficacy have an easier time adapting to the infant and in this study we hypothesised that the degree of self-efficacy also is associated with how difficult the mother believes it is to take care of the infant. The aim of this study was to investigate whether the late preterm infant’s mother’s self-efficacy in breastfeeding was associated with how the mother experienced her infant’s state-regulation at three months of corrected age.

**Methods:**

The study had a prospective and longitudinal design with a consecutive data collection through questionnaires. Inclusion criteria were mothers (*n* = 105) with a singleton infant born between 34 + 0 and 36 + 6 weeks of gestation. At term age, the mothers completed the Breastfeeding Self-efficacy Scale-Short Form and at the three months corrected age follow-up, mothers completed the Infant state-regulation index: questions related to whether the infant had difficulties with colic, persistent crying, comforting, falling asleep, sleep problems, breastfeeding, eating or poor weight gain.

**Results:**

The analyses showed that being an older mother, perceiving breastfeeding support, and having a higher breastfeeding self-efficacy were all significantly associated with identifying the infant as having better state-regulation.

**Conclusions:**

There was an association between mothers’ self-efficacy in breastfeeding and her perceptions of how good state-regulation the infant had. This is an important finding, as self-efficacy is a manageable factor that could positively affect how the mother perceives taking care of her infant. Clinical implication: Improved self-efficacy is known to be an important factor in increased breastfeeding prevalence and healthcare professionals should also target mother’s self-efficacy in breastfeeding to improve mother-infant relationship.

## Background

Breast milk is the best nutrition for both full-term (> 37 weeks) and preterm (< 37 weeks) infants [1] and is recommended by the World Health Organisation exclusively for the first six months and thereafter partially for two years or longer [2]. However, for various reasons, breastfeeding rates are lower among preterm infants compared to full term infants [3]. The preterm infant is often not mature enough to breastfeed exclusively after birth [4], and the mother does not always get the right support to establish exclusive breastfeeding because of institutional barriers to breastfeeding promotion [5]. These additional barriers can be maternal stress and anxiety [6]; the infant’s inability to breastfeed [7]; and suboptimal hospital routines, such as separation of mother and infant or late initiation of breast milk expression, as well as use of bottles, pacifiers and nipple shields [8].

The mother’s self-efficacy in breastfeeding is central to understanding which mothers are going to breastfeed their infants [9]. Persons with low self-efficacy often find tasks difficult to perform. If they fail, they blame themselves, whereas persons with high self-efficacy are prepared to test and try until they reach a solution. The Breastfeeding Self-Efficacy Scale-Short Form (BSES-SF) measures the mother’s self-efficacy in breastfeeding her infant [10]. The scale is validated in mothers of late preterm infants, and good breastfeeding support has been shown to strengthen the mother’s self-efficacy in breastfeeding [11]. It has previously been shown, in the same sample as in this study, that late preterm mothers who are more satisfied with the breastfeeding support given at hospital have higher BSES-SF scores and breastfeed for a longer period of time [11]. We have also shown, through development of the instrument Adaptation to the Late Preterm Infant when Breastfeeding Scale (ALPIBS), that a higher degree of self-efficacy was significantly associated with a higher degree of adaptation to the late preterm infant’s breastfeeding behaviour [12]. The ALPIBS is a 16-item instrument developed to measure mothers’ adaptation to their late preterm infant’s feeding behaviour. The questions are formulated as items describing mothers’ experience of adaptation to the infant in breastfeeding. Mothers with higher scores on ALPIBS have a more facilitating adaptation to the infant. This indicates that mothers with higher self-efficacy have an easier time adapting to the infant. We hypothesised that the degree of self-efficacy is also associated with how difficult the mother believes it is to take care of the infant. This assumption stems from the observation that mothers with poorer well-being perceive the infant as more often having problems with crying, sleeping and feeding, sometimes summarised as self-regulation difficulties [13], which is known as state-regulation difficulties among preterm infants [14]. For example, the mother’s anxiety is the best predictor of the infant’s temperament, resembling irritability and nursing difficulty [15], and mothers who rate their sleep as poor when their preterm infant is a newborn, more often perceive their infant as having sleeping difficulties later in life [16].

## Aim

The aim of this study was to investigate whether the late preterm infant’s mother’s self-efficacy in breastfeeding at term age was associated with how the mother experienced her infant’s state-regulation at three months of corrected age.

## Methods

### Design

The study had a prospective and longitudinal design with a consecutive data collection.

### Participants and data collection

Participants were recruited between September 2012 and July 2015 from a neonatal intensive care unit or maternity unit at a Swedish university hospital, after being identified through the logbook in the hospital delivery ward. Inclusion criteria were mothers with a singleton infant born between 34 + 0 and 36 + 6 weeks of gestation. Excluded participants and dropout rates of the eligible mother-infant pairs have been described elsewhere [11, 12]. The mothers were contacted by letter at term age (baseline; *n* = 148) and three months of corrected age (follow-up) and asked to complete paper-and-pencil questionnaires. For the present study, only the 110 mothers answering the infant state-regulation index at three months follow-up were eligible for participation. Moreover, since the focus of the study was on breastfeeding, we excluded five mothers who only fed their infant breast milk using a baby bottle. The study thus included a total of 105 breastfeeding mothers, after excluding 43 (29.1%) of the 148 mothers contacted at baseline.

### Study variables

At baseline (term age), the mothers completed the BSES-SF instrument, as well as a study-specific questionnaire containing questions about clinical and demographic characteristics, breastfeeding and infant formula use (Table 1). The BSES-SF instrument comprises 14 questions about how confident the mother is with breastfeeding her new baby, with answers given on a 5-point scale, ranging from “not at all confident” (1 point) to “very confident” (5 points). The total score on the instrument ranges from 14 to 70 points, with higher scores indicating better self-efficacy. The study-specific questions at baseline covered the mother’s age (years), if she was married/cohabiting (yes/no), had a college or university education (yes/no), was employed before giving birth (yes/no), used tobacco (yes/no), the number of children she had, if she was primiparas (yes/no), and if she perceived that she had support for breastfeeding from anyone in her vicinity (yes/no), in which case she was given the opportunity to specify who provided the support. Additionally, questions about the infant concerned if it was a vaginal birth (yes/no), the age of the infant (weeks), his or her gestational age (GA) at birth (weeks) and birthweight (grams), and if he or she was given breast milk only by breastfeeding (yes/no).

**Table 1:** Characteristics of the *n* = 105 participating mothers and their infants, together with results from the unadjusted linear regression analyses for predicting infant state-regulation at follow-up

Pertains to	Variable	Value	β (95% CI)	*P*-value
Mother	Age of mother at term age (years), mean (SD)	31.5 (4.8)	-0.150 (-0.247, -0.053)	**0.003**
	Married/Cohabiting, n (%)	104 (99.0)	-1.404 (-6.398, 3.591)	0.578
	College/University education, n (%)	48 (45.7)	0.527 (-0.442, 1.497)	0.283
	Employed before giving birth, n (%)	98 (93.3)	1.571 (-0.352, 3.495)	0.108
	Tobacco user, n (%)	1 (1.0)	-2.660 (-7.634, 2.313)	0.291
	Number of children, mean (SD)^a^	1.7 (0.8)	-0.962 (-1.520, -0.404)	**0.001**
	Primiparas, n (%)	54 (51.4)	1.185 (0.241, 2.129)	**0.014**
	Breastfeeding support, n (%)	79 (75.2)	-1.439 (-2.529, -0.349)	**0.010**
	Breastfeeding self-efficacy index, mean (SD)	56.6 (8.3)	-0.095 (-0.152, -0.038)	**0.001**
Infant	Vaginal birth, n (%)	81 (77.1)	0.358 (-0.797, 1.513)	0.540
	Age of infant at term age (weeks), mean (SD)	5.0 (3.9)	-0.019 (-0.145, 0.108)	0.772
	GA at birth (weeks), mean (SD)	36.0 (0.8)	-0.401 (-1.040, 0.238)	0.217
	Birthweight (kg), mean (SD)	2.76 (0.44)	-0.394 (-1.506, 0.718)	0.483
	Given breast milk only by breastfeeding, n (%)	90 (85.7)	0.711 (-0.670, 2.093)	0.310
Outcome	Infant state-regulation index, mean (SD)	2.6 (2.5)	-	-

All variables, except Infant state-regulation index, were measured at baseline (term age). There were 1 (1.0%) missing value for Tobacco user and 2 (1.9%) missing values for Breastfeeding self-efficacy and Age of infant at baseline

Significant *P*-values are given in bold

*CI* confidence interval, *GA* gestational age, *SD* standard deviation

^a^ Including the newborn infant

At the three months corrected age follow-up, mothers were asked to complete the Infant state-regulation index, comprising eight questions concerning the mothers’ perceptions of their infants’ ability to state regulate: these questions related to whether the infant had difficulties with colic, persistent crying, comforting, falling asleep, sleep problems, breastfeeding, eating or poor weight gain. Answers were given on a four-point scale, ranging from “no problems” (0 points) to “very severe problems” (3 points), which were then summed to produce a total score, ranging from 0 to 24 points, with high scores indicating more problems with state-regulation.

### Statistical analyses

Categorical data are presented as frequencies and percentages, *n* (%), while ordinal and continuous data are given as means with accompanying standard deviations (SDs). The associations between clinical, demographic, breastfeeding and infant formula use variables measured at baseline (term age) and the Infant state-regulation index measured at three months of corrected age follow-up were examined using adjusted and unadjusted linear regression analyses, with the results presented as slope coefficient β with accompanying 95% confidence intervals (CIs). Two different adjusted regression models are reported: a basic model including all variables having a *P*-value < 0.20 in the unadjusted regression analyses, and a trimmed model constructed by excluding variables with *P*-values ≥ 0.20 one-by-one from the basic model, starting with the variable with the highest *P*-value, and re-estimating the model until only variables with *P*-values < 0.20 remained in the model. All statistical analyses were performed using R 4.1.0 (R Foundation for Statistical Computing, Vienna, Austria), with two-sided *P*-values < 0.05 considered statistically significant.

### Ethics

Ethical approval for the study was given by the Regional Ethical Board at Uppsala University, Uppsala, Sweden (Dnr 210/287). Written material was given to the mothers, ensuring them of anonymity and the right to withdraw from participation at any time without giving any reason.

## Results

Characteristics of the *n* = 105 participating mothers and their infants are given in Table 1. At the time of answering the baseline questionnaire, the mothers had a mean (SD) age of 31.5 (4.8) years, with their infant being 5.0 (3.9) weeks old, having a GA at birth of 36.0 (0.8) weeks, and a birthweight of 2760 (438) grams. Almost all (*n* = 104; 99.0%) mothers were married or cohabiting, 48 (45.7%) had a college or university education, most (*n* = 98; 93.3%) were employed before giving birth, and almost no one (*n* = 1; 1.0%) was using tobacco. The mothers had a mean (SD) of 1.7 (0.8) children (including the newborn infant), with half (*n* = 54; 51.4%) of the mothers being primiparas, and 3 out of 4 mothers (*n* = 79; 75.2%) stating that they had support for breastfeeding from someone in their vicinity. The BSES-SF index among the 105 mothers was at a mean (SD) level of 56.6 (8.3) points, while they had a mean (SD) value of 2.6 (2.5) points on the Infant state-regulation index at three months follow-up. In total, 24 (22.9%) mothers had 0 points on the Infant state-regulation index and thus perceived that the infant had no difficulties at all with state-regulation.

### Who provided breastfeeding support?

Of the 79 mothers stating they had breastfeeding support, 75 (94.9%) specified who provided the support. The most common person the women perceived as providing support was their husband/partner (*n* = 60; 80%), followed by their own mother (*n* = 11; 14.7%). Additional answers included partner and mother (*n* = 2; 2.7%), family and friends (*n* = 1; 1.3%) and personnel (*n* = 1; 1.3%).

### Association with infant state-regulation index

Results from the linear regression analyses of the associations between clinical, demographic, breastfeeding and formula use variables measured at baseline (term age) and the Infant state-regulation index measured at three months corrected age follow-up are given in Table 1 for unadjusted linear regression analysis and Table 2 for adjusted linear regression analyses. In the unadjusted analyses, being an older mother, having more children, having breastfeeding support, and having a higher breastfeeding self-efficacy were all significantly associated with perceiving the infant as having better state-regulation, while being primiparas was significantly associated with perceiving the infant as having less state regulation. The same results were observed for the adjusted basic and trimmed linear regression analyses, except that number of children and being primiparas were not statistically significant anymore. Thus, for the trimmed model, for each additional year of the mother’s age, she perceived the infant to be 0.132 points better on the Infant state-regulation index (*P* = 0.009); furthermore, not having breastfeeding support was associated with 1.408 points lower value on the Infant state-regulation index (*P* = 0.006), while each additional point higher value on the BSES-SF index at term age was associated with the mother perceiving the infant to be 0.081 points better on the Infant state-regulation index at three months corrected age (*P* = 0.005).

**Table 2:** Results from the adjusted linear regression analyses for predicting infant state-regulation at follow-up

	Basic model		Final model	
Variable	β (95% CI)	*P*-value	β (95% CI)	*P*-value
Age of mother at term age (years)	-0.138 (-0.238, -0.037)	**0.008**	-0.132 (-0.231, -0.033)	**0.009**
Number of children^a^	-0.605 (-1.659, 0.449)	0.257	-0.451 (-1.061, 0.158)	0.145
Breastfeeding support	-1.333 (-2.346, -0.319)	**0.010**	-1.408 (-2.402, -0.414)	**0.006**
Breastfeeding self-efficacy index	-0.081 (-0.138, -0.024)	**0.006**	-0.081 (-0.137, -0.025)	**0.005**
Employed before giving birth	0.691 (-1.141, 2.523)	0.456	—	—
Primiparas	-0.403 (-2.050, 1.244)	0.628	—	—

Significant *P*-values are given in bold

*CI* confidence interval

^a^Including the newborn infant

## Discussion

The aim of this study was to investigate whether the late preterm infant’s mother’s self-efficacy in breastfeeding was associated with how the mother experienced her infant’s state-regulation at three months of corrected age. The results showed that being an older mother, perceiving breastfeeding support, and having a higher breastfeeding self-efficacy at infant’s term age were all significantly associated with identifying the infant as having better state-regulation at three months of corrected age. This is an important finding as both breastfeeding support and self-efficacy are manageable factors that could positively affect how difficult the mother perceives it to be to take care of the infant.

### Breastfeeding support and self-efficacy

Improved self-efficacy is known to be an important factor in increased breastfeeding prevalence. In a meta-analysis that included 11 articles, the authors stated that for each 1-point increase in the mean BSES-SF score, in the breastfeeding intervention group of mothers, the odds of exclusive breastfeeding increased by 10% [17]. This indicates that healthcare professionals should target mother’s self-efficacy in breastfeeding to improve breastfeeding rates.

### Preterm infant and state-regulation

**Fig. 1:**
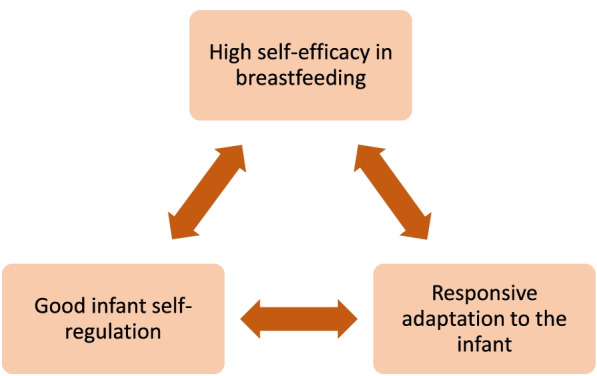
High self-efficacy creates a positive circle. The mother’s adaptation helps the infant in state-regulation

Preterm infants demonstrate more problems with state-regulation compared to term infants. They are more irritable, have problems with routines [14], and problematic feeding is highly prevalent [18]. The mother’s self-efficacy seems to have a mediating role in infant’s state-regulation. Infants whose mothers showed high levels of stress during pregnancy cried less if the mother also had high self-efficacy. We have previously shown that mothers with high self-efficacy have a more responsive adaptation to the infant during breastfeeding [12]. In this study, we have shown that high self-efficacy also has an impact on how mothers perceive the infant’s ability to state-regulate. Interaction between parents and infants can be described as the parent and infant responding to signals from the other and then sending signals back. The infant’s purpose with the communication is to keep the parent in close proximity [19]. Due to stress and anxiety, mothers of preterm infants often have problems adjusting to the parental role [20], and the infant is also more difficult to read and satisfy due to problems with state-regulation [14]. When the mothers cannot satisfy the infant’s needs, they may be filled with anxiety and guilt [21]. This could also be described as difficulties in attachment and bonding [19, 22]. Good breastfeeding support gives the mother higher self-efficacy in breastfeeding [17], and it may also facilitate attachment and bonding. A model of how high self-efficacy in breastfeeding affects the mother-infant relationship is provided in Fig. 1. As the figure shows, the connection between different concepts goes in both directions. Self-efficacy improves the mother’s adaptation to the infant, but the adaptation also strengthens the mother’s self-efficacy. The mother’s adaptation to the infant facilitates the infant’s state-regulation, and improved state-regulation facilitates the mother’s adaptation to her infant.

### The mother and breastfeeding support

Previous research has shown that mothers with older age are breastfeeding longer [23]. In this study, we have shown that mothers with older age perceive that the infant has better state-regulation. Another significant factor for how the mother perceived the infant’s state-regulation was that the mother perceived that she had breastfeeding support. By far, the most common person that the women perceived as providing support was their husband/partner. Research has suggested that the most effective breastfeeding support from the father is delivered using a sensitive, coordinated teamwork approach that is responsive to the mother’s needs [24].

### Strengths and limitations

Among the strengths of the present study was the prospective design, whereby the predictors were measured three months before the outcome, meaning that we could ascertain that the Infant state-regulation index score could not influence the BSES-SF score, but any possible influence had to go the other way around. A limitation of the study was that data collection was performed in a university city with a high level of education. For this reason, data cannot be generalised to regions with lower levels of education. A further limitation was that the Infant state-regulation index outcome was study-specific and has not been psychometrically validated formally.

## Conclusions

Breastfeeding self-efficacy is a manageable factor that reflects mothers’ confidence in breastfeeding. In this study there was an association between mothers’ self-efficacy in breastfeeding and how good state-regulation the mother experienced that the infant had. This is an important finding, as self-efficacy is a factor that healthcare professionals can target to improve breastfeeding rates, but also and at the same time, positively affect how difficult the mother perceives it to be to take care of her infant.

## Data Availability

The datasets used and/or analysed during the current study are available from the corresponding author upon reasonable request.

## Abbreviations

BSES-SF: Breastfeeding Self-efficacy Scale-Short Form
CI: Confidence interval
GA: Gestational age
SD: Standard deviation
